# Population structure of the *Monocelis lineata* (Proseriata, Monocelididae) species complex assessed by phylogenetic analysis of the mitochondrial Cytochrome c Oxidase subunit I (*COI*) gene

**DOI:** 10.1590/S1415-47572009005000076

**Published:** 2009-12-01

**Authors:** Daria Sanna, Tiziana Lai, Paolo Francalacci, Marco Curini-Galletti, Marco Casu

**Affiliations:** Dipartimento di Zoologia e Genetica Evoluzionistica, University of Sassari, SassariItaly

**Keywords:** microturbellaria, sequencing, sibling species, phylogeography, phylogeny

## Abstract

*Monocelis lineata* consists of a complex of sibling species, widespread in the Mediterranean and Atlantic Ocean. Previous genetic analysis placed in evidence at least four sibling species. Nevertheless, this research was not conclusive enough to fully resolve the complex or to infer the phylogeny/phylogeography of the group. We designed specific primers aiming at obtaining partial sequences of the mtDNA gene Cytochrome c Oxidase subunit I (*COI*) of *M. lineata*, and have identified 25 different haplotypes in 32 analyzed individuals. The dendrogram generated by Neighbor-Joining analysis confirmed the differentiation between Atlantic and Mediterranean siblings, as well as the occurrence of at least two Mediterranean sibling species. Thus validated, the method here presented appears as a valuable tool in population genetics and biodiversity surveys on the *Monocelis lineata* complex.

*Monocelis lineata* (O.F. Müller, 1774) (Proseriata: Monocelididae) is a flatworm (Platyhelminthes) characterized by a comparatively “simple” morphology, and a wide distribution along the north Atlantic, Mediterranean and Black Sea coasts, occurring in brackish-water and marine habitats on any kind of substrate (Ax, 1956). Across this range, the species shows a remarkably uniform morphology, except for polymorphism related to the ocular pigment shield, which may be absent in the entire population (Curini-Galletti and Mura, 1998; Casu and Curini-Galletti, 2004). Previous molecular assays, carried out by using allozyme electrophoresis, suggested that the taxon consists of at least four sibling species, three Mediterranean species with a sharp genetic separation between brackish-water (with pigmented eyespots) and marine (unpigmented) populations, and one genetically heterogeneous, as yet unresolved “sibling” in the north Atlantic (Casu and Curini-Galletti, 2004). However, even though allozymes have proved, in past years, to be a powerful tool in discriminating sibling complexes (Manchenko and Radashevsky, 1998; Klautau *et al.*, 1999; De Matthaeis *et al.*, 2000; Maltagliati *et al.*, 2000, for marine invertebrates), their application is biased by certain technical limitations (among others, the scarce reproducibility across different laboratories), which hinder routine use of the marker in biodiversity surveys. On the contrary, the sequencing of mitochondrial DNA (mtDNA) gene coding for Cytochrome c Oxidase subunit I (*COI*) is usually performed to arrive at inferences on phylogeny and/or phylogeography in different species (Breton *et al.*, 2003, for marine invertebrates). However, so far *COI* sequencing has not been applied to studies on interstitial micro-turbellarians.

We designed specific primers to amplify a partial region of *COI* in *M. lineata*, and validated it, while studying Mediterranean and Atlantic specimens, as a tool for population genetic studies and biodiversity surveys on the *Monocelis lineata* complex.

In a first step, universal primers for marine invertebrates (Folmer *et al.*, 1994) were tested on 160 individuals from 32 populations from the northeastern Atlantic and western to eastern Mediterranean (about five specimens per sampling site) (Figure 1). We aimed at obtaining at least one suitable sequence to use as a base for designing specific primers. Specimens had been stored in 70% alcohol for a period of two to five years. DNA was extracted from the entire individual using the DNeasy® Tissue Kit (QIAGEN Inc.). PCR amplification was carried out in 25 μL total volume, containing about 5 ng/μL of total genomic DNA, 0.4 μM of each primer, 2.5 U of Taq polymerase (Euro Taq®, Euroclone), 1.25 mM MgCl_2_, in a reaction mix containing 200 μM of dNTP mix and 1 x buffer. The PCR profile consisted of an initial hot start step (2 min at 94 °C), and 35 cycles, each comprising denaturation for 1 min at 94 °C, annealing for 1 min at 52 °C and extension for 1.30 min at 72 °C, followed by a final extension for 5 min at 72 °C. For checking products obtained with universal primers, samples were electrophoresed on 2% agarose/0.5 x TBE gels stained with ethidium bromide (10 mg/mL), at 4 V/cm for 20 min. Purified PCR products (ExoSAP-IT®,USB Corporation,) were cycle-sequenced using the BigDye Sequencing Kit, Terminator 3.1® (Applied Biosystems), and then analyzed on a 3100 ABI PRISM Avant® (Applied Biosystems) automated sequencer. Cycle-sequencing reactions were carried out in 10 μL total volume, containing 2 to 6 μL of purified PCR product and 0.32 μM of the forward or the reverse primer. An initial hot start step (1 min at 96 °C) was followed by 35 cycles, each comprising denaturation (10 s at 96 °C), annealing (5 s at 50 °C) and extension (4 min at 60 °C). Cycle-sequencing products were purified using the SigmaSpin Post-Reaction Clean-Up Columns® (Sigma Aldrich). Only three from the Mediterranean populations (SGo, MZo, and PPx; Figure 1) among the 160 specimens tested with universal primers showed satisfactory amplification of the target region. Sequences of 577 bp, 568 bp, and 657 bp corresponding to GenBank accession numbers EF583451, EF591057, and EU889254, respectively, were obtained. Using the software Mega 4 (Tamura *et al.*, 2007), these sequences were aligned to those of the Platyhelminthes *Nematoplana coelogynoporoides* (Proseriata: Nematoplanidae) (GenBank accession number: AJ405985), and *Vannuccia* sp. (Proseriata: Coelogynoporidae) (GenBank accession number: AJ405986). Nucleotide alignment disclosed a high degree of identity, about 69% for *N. coelogynoporoides* and 68% for *Vannuccia* sp., thus pointing to the correct amplification of *COI* in *M. lineata*. Specific internal primers have been designed within the most conserved regions using Primer Premier 5.00 software (PREMIER Biosoft International, Palo Alto, CA, Table 1). The above-described PCR conditions, except for the annealing temperature at 51 °C, were used for the designed primers, thus permitting amplification of a 195-199 bp fragment of the *COI* gene in three specimens of *M.lineata*. We then studied one individual from each sampling site (Figure 1) in a total of 32 specimens, in order to investigate genetic variability between and within Atlantic and Mediterranean populations.

PCR amplification with the designed primers yielded short fragments (about 200 bp) of the *COI* gene. This however does not represent a bias, for it has been demonstrated that sequences of about or less than two hundred bp may correctly show the phylogenetic/phylogeographic traits of the species (Tillier *et al.*, 1992; Kirby and Reid, 2001, Bucklin and Allen, 2004, Hajibabaei *et al.*, 2006).

The results obtained by using DNAsp 4.0 software (Rozas *et al.*, 2003) revealed a low mean nucleotide value (*Pi* = 0.21) and elevated mean haplotype diversity (*Hd* = 0.97), distributed on 25 diverse haplotypes out of the 32 sequences analysed (GenBank accession numbers: EU889254-EU889265; EU889268-EU889272; EU889275-EU889276; EU889278-EU889291). The Neighbor-Joining consensus dendrogram (Figure 2), constructed by means of MEGA4 (Tamura *et al.*, 2007) after 1,000 bootstrap replicates and by applying the Maximum composite likelihood method (MCL), placed in evidence the sharp separation between Atlantic and Mediterranean samples. Furthermore, within the Mediterranean group itself, two main clusters were observed (Figure 2), with individuals from brackish-waters (with the ocular pigment shield) and marine waters (without the ocular pigment shield), respectively. Main nodes were supported by bootstraps higher than 78% (Figure 2). Although preliminary, these findings, consistent with previous allozyme data (Casu and Curini-Galletti, 2004) showing conspicuous genetic differentiation between Atlantic and Mediterranean, and among Mediterranean populations themselves, further support the occurrence of a *M. lineata* sibling species complex.

**Figure 1:**
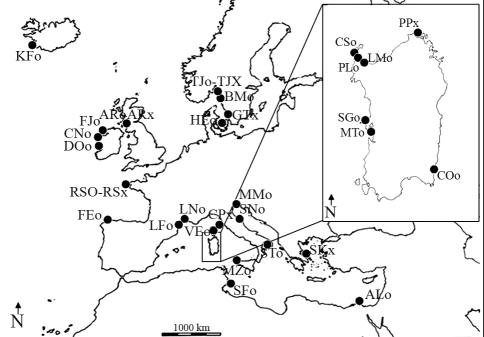
Sampling sites. In the sample three-letter code, “o” indicates the presence of ocular pigment shield, “x” its absence. Iceland, KFo: Keflavik. Sweden, GTx: Göteborg; BMo: Bohus Malmön; TJo-TJx: Tjarnö. Denmark, HEo: Helsingør. Scotland, ARo-ARx: Ardrossan. Ireland, FJo: Fjord; DOo: Doolin; CNo: Connemara. Spain, FEo: Ferrol. France, RSo-RSx: Roscoff; LNo: Port La Nouvelle; LFo: La Franqui; VEo, Ventilegne. Italy, CSo: Stagno di Casaraccio; PLo: Stagno di Pilo; LMo: Porto Torres; SGo: Santa Giusta; MTo: Stagno di Mistras; PPx: Porto Pozzo; COo: Stagno di Colostrai; CPx: Castiglione della Pescaia; MZo: Mozia; MMo: Miramare; SNo: Senigallia; STo: Porto Cesareo. Greece, SKx: Skopelos. Tunisia, SFo: Sfax. Egypt, ALo: El-Mountazah, Alexandria.

**Figure 2:**
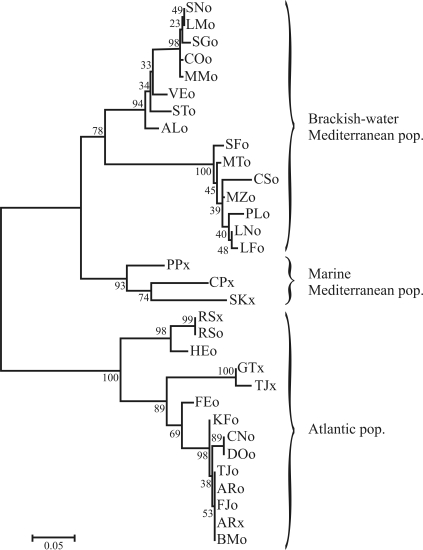
MCL Neighbor-Joining dendrogram for the *COI* fragment of the sampled specimens of *M. lineata*. Bootstrap values are shown above the branches. The location codes are the same as in Figure 1.

In this context, the newly designed primers can be adequately used to obtain sequences from individuals of *M. lineata*, with high haplotypic diversity and a low level of nucleotide variability. Furthermore, the high reproducibility of the technique allows for increasing the number of individuals/populations over time. The primers here reported may thus be confidently used to resolve the complex of sibling species in *M. lineata*, and to depict phylogeographic patterns within each sibling, on both a regional and local scale. Indeed, *COI* has been routinely used to successfully distinguish cryptic species in different “simple” organisms, *e.g.* Anisakid nematodes (Hu *et al.*, 2001; Cross *et al.*, 2006; Derycke *et al.*, 2007).

Furthermore, the problems for non-specialists concerning the correct identification of minute mesopsammic organisms have always been a hindrance in the use of the meiofauna in ecological surveys and in assessing the actual extent of local biodiversity (Kennedy and Jacoby, 1999). Therefore, in the light of the present trend for reducing taxonomic expertise (the so-called taxonomic impediment, Boero, 2001), the finding of suitable primers for *COI* may be an invaluable contribution for future researches. Indeed, DNA barcoding (for a review, see Moritz and Cicero, 2004) by means of *COI* sequencing has been suggested as a promising tool to assess the actual level of marine biodiversity.

## Figures and Tables

**Table 1:** *COI* primer sequence, GenBank accession number and size of the sequences obtained for each sample of *Monocelis lineata* analysed.

Locus	Primer sequences (5'-3')	GenBank accession n.	Size (bp)	Sample
		EU889255	195	ALo
		EU889256	198	COo
		EU889257	198	SGo
		EU889258	198	STo
		EU889259	198	MMo
		EU889260	199	MZo
		EU889261	198	CSo
		EU889262	198	LNo
		EU889263	198	MTo
		EU889264	198	PLo
		EU889265	198	PPx
		EU889268	198	CPx
		EU889269	198	SKx
		EU889270	198	VEo
		EU889271	198	SNo
*COI*	L: GTAATGCCDGTDCTTTTTGGAGG H: CTHACCCCWGCCAAATGTAAA	EU889272	198	LMo
		EU889275	198	SFo
		EU889276	198	LFo
		EU889278	195	CNo
		EU889279	195	DOo
		EU889280	195	HEo
		EU889281	195	FEo
		EU889282	195	RSx
		EU889283	195	RSo
		EU889284	195	GTx
		EU889285	195	ARo
		EU889286	195	ARx
		EU889287	195	FJo
		EU889288	195	KFo
		EU889289	195	BMo
		EU889290	195	TJo
		EU889291	198	TJx

L, light chain; H, heavy chain.
